# Supplementary material for: A UMLS-based spell checker for natural language processing in vaccine safety
Source: BMC Med Inform Decis Mak. 2007 Feb 12;7:3. doi: 10.1186/1472-6947-7-3 (PMC1805499; doi:10.1186/1472-6947-7-3)
Supplement: Additional file 13 — RAP application directory. Sets up directory for RAP (RDF application for PHP) source code [file 1472-6947-7-3-S13.gz › rap/doc/phpdoc/ontModel/OWLVocabulary.html]

xml version="1.0" encoding="iso-8859-1"?


Docs For Class OWLVocabulary


## Class OWLVocabulary

Description

Description |
Methods (details)

OWL vocabulary items

History: <LI>10-05-2004 : First version of this class.</LI>

- access: public
- author: Daniel Westphal <at d-westphal dot de mail at d-westphal dot de>
- version: V0.9.1

Located in /ontModel/OWLVocabulary.php (line 23)

```
OntVocabulary
   |
   --OWLVocabulary
```

Method Summary

Description |
Methods (details)

void
ALL\_DIFFERENT
()

void
ALL\_VALUES\_FROM
()

void
ANNOTATION\_PROPERTY
()

void
BACKWARD\_COMPATIBLE\_WITH
()

void
CARDINALITY
()

object ResProperty
COMMENT
()

void
COMPLEMENT\_OF
()

void
DATATYPE
()

void
DATATYPE\_PROPERTY
()

void
DATATYPE\_RESTRICTION
()

void
DATA\_RANGE
()

void
DEPRECATED\_CLASS
()

void
DEPRECATED\_PROPERTY
()

void
DIFFERENT\_FROM
()

void
DISJOINT\_WITH
()

void
DISTINCT\_MEMBERS
()

object ResProperty
DOMAIN
()

void
EQUIVALENT\_CLASS
()

void
EQUIVALENT\_PROPERTY
()

void
FUNCTIONAL\_PROPERTY
()

void
HAS\_VALUE
()

void
IMPORTS
()

void
INCOMPATIBLE\_WITH
()

void
INTERSECTION\_OF
()

void
INVERSE\_FUNCTIONAL\_PROPERTY
()

void
INVERSE\_OF
()

object ResProperty
IS\_DEFINED\_BY
()

object ResProperty
LABEL
()

void
MAX\_CARDINALITY
()

void
MIN\_CARDINALITY
()

void
NAMESPACE
()

void
NOTHING
()

void
OBJECT\_CLASS
()

void
OBJECT\_PROPERTY
()

void
OBJECT\_RESTRICTION
()

void
ONE\_OF
()

object ResResource
ONTCLASS
()

void
ONTOLOGY
()

void
ON\_PROPERTY
()

void
PRIOR\_VERSION
()

void
PROPERTY
()

object ResProperty
RANGE
()

void
RESTRICTION
()

void
SAME\_AS
()

void
SAME\_CLASS\_AS
()

void
SAME\_INDIVIDUAL\_AS
()

void
SAME\_PROPERTY\_AS
()

object ResProperty
SEE\_ALSO
()

void
SOME\_VALUES\_FROM
()

object ResProperty
SUB\_CLASS\_OF
()

object ResProperty
SUB\_PROPERTY\_OF
()

void
SYMMETRIC\_PROPERTY
()

void
THING
()

void
TRANSITIVE\_PROPERTY
()

object ResProperty
TYPE
()

void
UNION\_OF
()

void
VERSION\_INFO
()

Methods

Description |
Methods (details)

ALL\_DIFFERENT (line 133)

void

ALL\_DIFFERENT
()

ALL\_VALUES\_FROM (line 138)

void

ALL\_VALUES\_FROM
()

ANNOTATION\_PROPERTY (line 128)

void

ANNOTATION\_PROPERTY
()

BACKWARD\_COMPATIBLE\_WITH (line 143)

void

BACKWARD\_COMPATIBLE\_WITH
()

CARDINALITY (line 148)

void

CARDINALITY
()

COMMENT (line 55)

Answer the predicate that denotes comment annotation on an ontology element.

- access: public

object ResProperty

COMMENT
()

COMPLEMENT\_OF (line 153)

void

COMPLEMENT\_OF
()

DATATYPE (line 158)

void

DATATYPE
()

DATATYPE\_PROPERTY (line 163)

void

DATATYPE\_PROPERTY
()

DATATYPE\_RESTRICTION (line 173)

void

DATATYPE\_RESTRICTION
()

DATA\_RANGE (line 168)

void

DATA\_RANGE
()

DEPRECATED\_CLASS (line 178)

void

DEPRECATED\_CLASS
()

DEPRECATED\_PROPERTY (line 183)

void

DEPRECATED\_PROPERTY
()

DIFFERENT\_FROM (line 193)

void

DIFFERENT\_FROM
()

DISJOINT\_WITH (line 198)

void

DISJOINT\_WITH
()

DISTINCT\_MEMBERS (line 188)

void

DISTINCT\_MEMBERS
()

DOMAIN (line 43)

Answer the predicate that denotes the domain of a property.

- access: public

object ResProperty

DOMAIN
()

EQUIVALENT\_CLASS (line 203)

void

EQUIVALENT\_CLASS
()

EQUIVALENT\_PROPERTY (line 208)

void

EQUIVALENT\_PROPERTY
()

FUNCTIONAL\_PROPERTY (line 213)

void

FUNCTIONAL\_PROPERTY
()

HAS\_VALUE (line 218)

void

HAS\_VALUE
()

IMPORTS (line 228)

void

IMPORTS
()

INCOMPATIBLE\_WITH (line 223)

void

INCOMPATIBLE\_WITH
()

INTERSECTION\_OF (line 233)

void

INTERSECTION\_OF
()

INVERSE\_FUNCTIONAL\_PROPERTY (line 238)

void

INVERSE\_FUNCTIONAL\_PROPERTY
()

INVERSE\_OF (line 243)

void

INVERSE\_OF
()

IS\_DEFINED\_BY (line 66)

Answer the predicate that denotes isDefinedBy annotation on an ontology element

- access: public

object ResProperty

IS\_DEFINED\_BY
()

LABEL (line 77)

Answer the predicate that denotes label annotation on an ontology element

- access: public

object ResProperty

LABEL
()

MAX\_CARDINALITY (line 248)

void

MAX\_CARDINALITY
()

MIN\_CARDINALITY (line 253)

void

MIN\_CARDINALITY
()

NAMESPACE (line 359)

void

NAMESPACE
()

NOTHING (line 258)

void

NOTHING
()

OBJECT\_CLASS (line 263)

void

OBJECT\_CLASS
()

OBJECT\_PROPERTY (line 268)

void

OBJECT\_PROPERTY
()

OBJECT\_RESTRICTION (line 273)

void

OBJECT\_RESTRICTION
()

ONE\_OF (line 278)

void

ONE\_OF
()

ONTCLASS (line 32)

Answer the resource that represents the class 'class' in this vocabulary.

- access: public

object ResResource

ONTCLASS
()

ONTOLOGY (line 288)

void

ONTOLOGY
()

ON\_PROPERTY (line 283)

void

ON\_PROPERTY
()

PRIOR\_VERSION (line 294)

void

PRIOR\_VERSION
()

PROPERTY (line 299)

void

PROPERTY
()

RANGE (line 88)

Answer the predicate that denotes the domain of a property.

- access: public

object ResProperty

RANGE
()

RESTRICTION (line 304)

void

RESTRICTION
()

SAME\_AS (line 309)

void

SAME\_AS
()

SAME\_CLASS\_AS (line 314)

void

SAME\_CLASS\_AS
()

SAME\_INDIVIDUAL\_AS (line 319)

void

SAME\_INDIVIDUAL\_AS
()

SAME\_PROPERTY\_AS (line 324)

void

SAME\_PROPERTY\_AS
()

SEE\_ALSO (line 99)

Answer the predicate that denotes seeAlso annotation on an ontology element

- access: public

object ResProperty

SEE\_ALSO
()

SOME\_VALUES\_FROM (line 329)

void

SOME\_VALUES\_FROM
()

SUB\_CLASS\_OF (line 110)

Answer the predicate that denotes that one class is a sub-class of another.

- access: public

object ResProperty

SUB\_CLASS\_OF
()

SUB\_PROPERTY\_OF (line 121)

Answer the predicate that denotes that one property is a sub-property of another.

- access: public

object ResProperty

SUB\_PROPERTY\_OF
()

SYMMETRIC\_PROPERTY (line 334)

void

SYMMETRIC\_PROPERTY
()

THING (line 339)

void

THING
()

TRANSITIVE\_PROPERTY (line 344)

void

TRANSITIVE\_PROPERTY
()

TYPE (line 370)

Answer the predicate that denotes the rdf:type property.

- access: public

object ResProperty

TYPE
()

UNION\_OF (line 349)

void

UNION\_OF
()

VERSION\_INFO (line 354)

void

VERSION\_INFO
()

Documentation generated on Thu, 7 Jul 2005 13:42:37 +0200 by phpDocumentor 1.3.0RC3
